# Rituximab induced lung injury

**DOI:** 10.1186/s12890-025-03802-x

**Published:** 2025-07-28

**Authors:** Rohit Chari, Youmna Abdelghany, Madeleine Purcell, Blaine Kenaa

**Affiliations:** 1 University of Maryland School of Medicine, Baltimore, MD USA; 2 Department of Medicine, Division of Pulmonary and Critical Care Medicine, University of Maryland School of Medicine, 110 S Paca Street, 2nd Floor, Baltimore, MD USA

**Keywords:** Rituximab, Interstitial lung disease, Chemotherapy toxicity

## Abstract

**Background:**

Rituximab is a chimeric human-mouse immunoglobulin monoclonal antibody with high affinity for CD20 surface antigens expressed by pre-B and B cells that is commonly used as the mainstay for the treatment of B cell non-Hodgkin’s lymphomas, including diffuse large B-cell lymphoma (DLBCL). As the drug has become more widely used, rituximab associated Interstitial Lung disease (RTX-ILD) is being recognized as potential complication (Non-infectious pulmonary toxicity of rituximab: a systematic review| Rheumatology| Oxford Academic).

**Case report:**

We discuss a 73-year-old woman with newly diagnosed DLBCL who underwent chemotherapy and immunotherapy with rituximab, cyclophosphamide, doxorubicin hydrochloride, vincristine sulphate, and prednisone (R-CHOP). Following her initial rituximab infusion, she developed shortness of breath, chills, rigors, flushing, and agitation. The rituximab infusion was paused, and hypersensitivity reaction medications were given per protocol. The infusion was resumed at a slower rate. Two weeks after initial infusion, she was hospitalized for shortness of breath and hypoxemia to 88% on pulse oximeter requiring 2 L of nasal cannula oxygen. Chest imaging showed new diffuse ground glass opacities (GGOs) on top of apical scarring, upper lobe emphysema, and few calcified granulomas. Patient underwent bronchoscopy for bronchoalveolar lavage (BAL) which was negative for infections and malignancy. Given the temporal relationship, chemotherapy induced lung injury was high on the differential, with rituximab being the possible offending agent. She was started on prednisone 60 milligram for 5 days with a follow up chest imaging showing resolution of the acute GGO. Her O2 requirements decreased from 3 L to 1 L and she was sent home with oxygen. Given the curative intent of R-CHOP, after shared decision making with the patient and her medical team, a treatment plan with a longer course of high and low prednisone was incorporated as part of her chemotherapy session. She was able to successfully finish her treatment with no additional episode, at which point she was able to be successfully tapered off her prednisone.

**Discussion:**

Rituximab induced ILD is rare but given its severity requires a high index of suspicion for diagnosis. Given the potential for long term complication, once suspected, treatment should be discontinued. Here we detail how a prolonged steroid course could be used as adjunct therapy of ILD if therapy with rituximab is considered curative and essential.

**Conclusion:**

Rituximab and Cyclophosphamide are well described causes of acute pneumonitis post R-CHOP administration. Given curative effect of R-CHOP, careful changes in management plan and co-treatment with steroids could help preserve lung function while allowing for full continuation of the chemotherapy regimen.

## Introduction

Rituximab is a chimeric human-mouse immunoglobulin monoclonal antibody with high affinity for CD20 surface antigens expressed by pre-B and B cells [2]. As the first therapeutic immunotherapy approved by the food, drug administration (FDA) in 1997, it has improved outcomes in the treatment of B-cell malignancies including follicular lymphoma, chronic lymphocytic leukemia and non-Hodgkin’s lymphomas, including diffuse large B-cell lymphoma (DLBCL) [2–4]. Though most patients tolerate the drug with minimal side-effects, infusion related hypersensitivity reaction is seen in 9–15% of patients as well as anaphylaxis and Acute Respiratory Distress Syndrome (ARDS) in rare occasions [5]. Despite its success in the treatment of hematologic and rheumatologic diseases, more recent reviews have described respiratory events up to 38% with specific rituximab associated interstitial lung disease (RTX-ILD) ranging from 3–10%^1,6,7^. We describe a case of rituximab associated lung injury in a patient with DLBCL that was successfully treated with a longer course of steroids while allowing concomitant administration of rituximab.

## Case presentation

A 73-year-old woman with a recent diagnosis of DLBCL presented to the emergency department with a four-day history of worsening shortness of breath and hypoxemia 88% on room air requiring 2–3 L supplemental oxygen. Her social history includes a 25-pack year of tobacco smoking which she quit 33 years before her diagnosis. Pertinent family history includes breast cancer in her mother and sister. The patient has received both sars-cov-2 and influenza vaccination for the year.

Two weeks prior to her current presentation, patient had received the first cycle of rituximab, cyclophosphamide, doxorubicin, vincristine and prednisolone (R-CHOP). Her initial infusion was complicated by an episode of lacy, erythematous rash that was attributed to potential hypersensitivity reaction. A week after administration, she was noted to have persistent dyspnea associated with small pleural effusion and lower extremity edema. She was started on a diuretic for concern for possible volume overload with a close follow up in a week. No thoracentesis was done given small size and no safe window.

Upon presentation to the emergency department, she was visibly dyspneic at rest and with minimal exertion. Initial vitals showed a normal temperature of 36.9 °C, blood pressure, 124/75 and heart rate at 100 beats per minute. On physical examination, her pulmonary auscultation revealed diffuse rhonchi with decreased breath sounds at the left lower lung base and 1 + putting edema in the lower extremity.

**Fig. 1:**
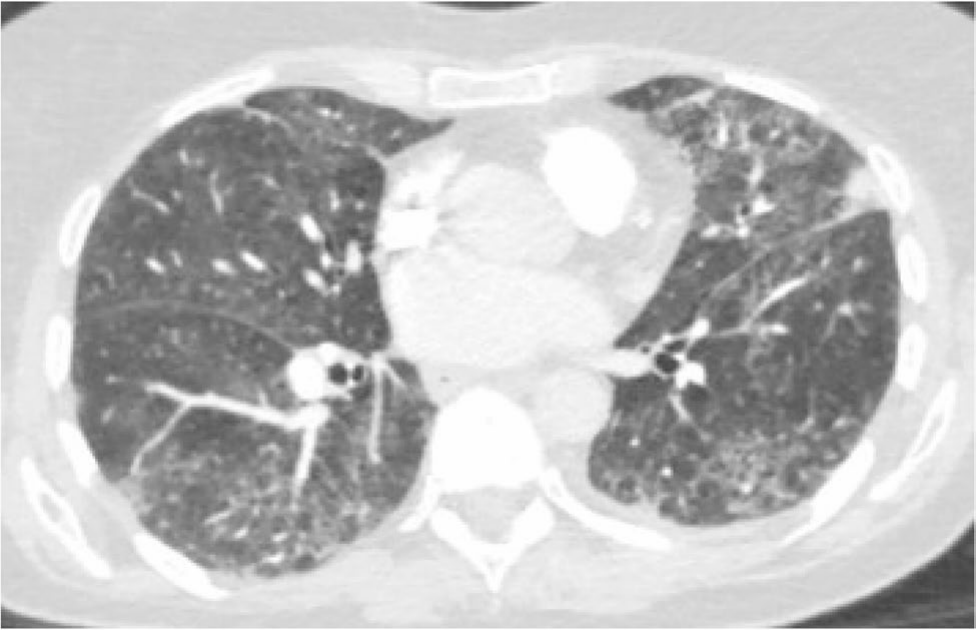
CT chest prior to treatment showing diffuse ground glass opacities

Initial work up in the emergency room included complete blood count (CBC), comprehensive metabolic panel (CMP), Troponin, prothrombin (PT) and international normalized ration (INR), Urinalysis, thyroid stimulating hormone (TSH), electrocardiogram (EKG), chest radiography (chest x-ray), and computed tomography angiography (CTA) of the chest. EKG showed premature atrial complexes, inferior infarct, and a shortened QT interval. Chest x-ray revealed a left perihilar opacity and a small left pleural effusion. Chest tomography angiogram of the chest revealed no pulmonary embolism, multifocal ground glass opacities (GGO) present in all five lobes, emphysema, and trace left pleural effusion with atelectasis (Fig. 1). Labs demonstrated mildly elevated white blood cell (WBC) count.

Further work up once admitted to hospital showed an elevated lactate dehydrogenase (LDH), positive antinuclear antibody with a 1:80 titer and homogenous pattern, negative urine legionella, urine strep pneumonia, negative respiratory viral panel, and negative SARS-COV-2 RNA test. Anti-double-stranded deoxyribonucleic acid antibody (dsDNA) was also positive at 75. Pulmonology and infectious disease were consulted for further work up of hypoxemia and concern for infection. Bronchoscopy was performed and revealed a normal tracheobronchial tree, negative airway erythema or edema, and mild tracheomalacia. Right middle lobe (RML) bronchoalveolar lavage (BAL) studies including respiratory culture, Herpes simplex virus 1/2 polymerase chain reaction (PCR), Cytomegalovirus PCR, toxoplasma PCR, adenovirus PCR, pneumocystis jirovecii (PJP) direct fluorescence antibody (DFA), legionella culture, nocardia culture, fungal, acid fast bacilli (AFB) cultures and cytology were all negative. A biopsy wasn’t performed given underlying hypoxemia and background of emphysema on CT of the chest. Pulmonary function tests revealed moderately obstructive ventilatory defect, moderate restrictive ventilatory defect, and severe gas transfer defect. Transthoracic echocardiogram (TTE) was also performed and showed a normal ejection fraction without valvular abnormalities.

The differential for this immunocompromised patient with acute hypoxemic respiratory failure and imaging showing multi-lobar ground glass opacities is broad and includes hydrostatic pulmonary edema, atypical infections, lymphatic metastasis, auto immune etiologies with pulmonary involvement, and drug induced lung injury.

Given the evidence of volume overload on physical exam, pulmonary edema was initially high on the differential, however she had minimal clinical or radiological improvement with diuretic treatment. Her non-invasive infectious work up was negative making viral or atypical bacterial infection unlikely. Pt was also on levofloxacin due to history of neutropenia making common bacterial infection less likely. Due to the patient’s immunocompromised status, opportunistic infections such as PJP, fungal infections such as histoplasmosis and blastomycosis were also possible causes of hypoxia. Our patient lived in Maryland and recently travelled to North Carolina, making histoplasmosis and blastomycosis possible causes of infection based on geography. Parasitic infection was a possible but unlikely cause of hypoxia and bilateral GGOs as our patient did not have an eosinophilia. Bronchoscopy was performed with negative fungal stain and silver stain ruling out PJP and fungal infections. There were also no signs of parasitic infection on bronchoscopy. Metastatic disease was also initially on the differential; however, CT chest was not consistent with lymphangitic carcinomatosis. Autoimmune diseases including Systemic lupus erythematous (SLE) was also considered given positive ANA and dsDNA. Pulmonary manifestation [6] of SLE can present as pneumonitis and diffuse alveolar hemorrhage contributing towards patients mortality. However given the lack of hemorrhage on BAL and other associated systemic symptoms, it was considered less likely.

Given exclusion of other etiologies, we were highly concerned about drug induced lung injury due to chemotherapy. Though multiple drugs can cause lung injury, the acuity, timeline and prior hypersensitivity reaction to the drug raised concern for Rituximab associated lung injury as similar cases were described in the literature. While Cyclophosphamide was also a consideration, given the systemic reaction to Rituximab and onset shortly after, it was thought to be less likely than Rituximab.

**Fig. 2:**
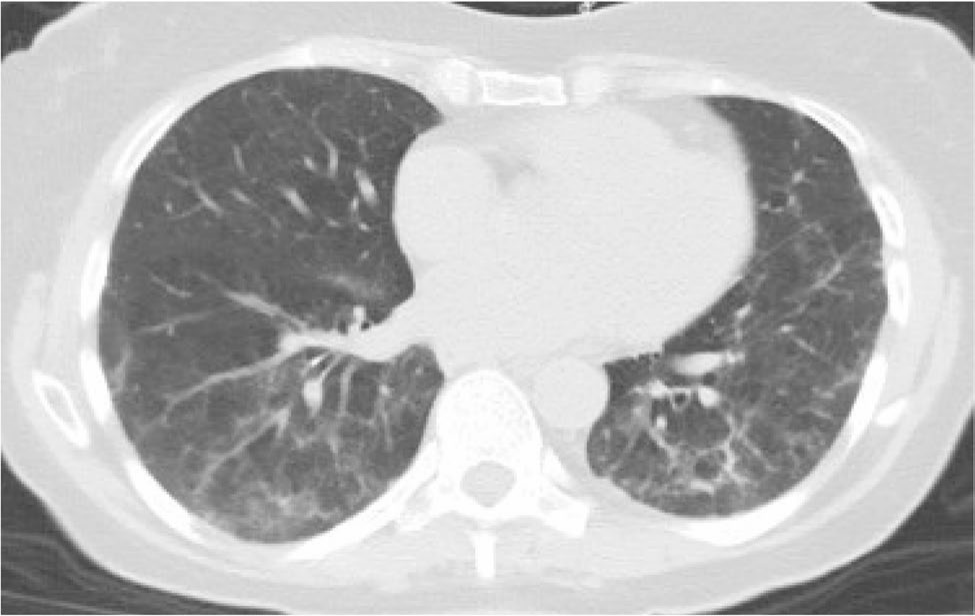
Chest post treatment showing improvement of ground glass opacities

Due to patient’s hypoxemia, she was continued on 2 L nasal cannula (NC). She was initially started on ceftriaxone 1 g q24h and azithromycin 500 mg q24h for treatment of community acquired pneumonia (CAP). She was continued on her home acyclovir 800 mg twice a day for HSV prophylaxis. The infectious disease team was consulted who recommended stopping CAP treatment. Due to concern for drug induced lung injury (DILI), she was started on prednisone 1 mg/kg (60 mg daily) for 5 days. Repeat CT after 5 days showed interval improvement of GGOs (Fig. 2). Pt went from requiring 2 L NC at rest to 1 L NC and was eventually weaned off supplemental oxygen. Extensive discussions were held between the oncology team, pulmonary team, patient and her family on the cause of this lung injury most likely being related to chemotherapy. The patient and her family decided to continue the entirety of R-CHOP therapy given the curative intent with limited alternative treatment options with plan of continuing steroids beyond the standard 100 mg of prednisone course that is part of R-CHOP regimen. On the day of rituximab infusion, her steroid dose was increased to 100 mg daily which was continued for 5 days and then tapered down to 40 mg maintenance until next cycle along with a trimethoprim/sulfamethoxazole for Pneumocystis Jirovecii pneumonia (PJP) prophylaxis with a plan to continue for a total of four additional cycles which would complete her chemotherapy.

Following discharge, patient followed up with pulmonary outpatient and was able to tolerate and complete R-CHOP course without additional complications. Regarding the prednisone course, following the third cycle of R-CHOP therapy the maintenance dose was reduced from 40 mg of prednisone to 20 mg prednisone daily given improvement of CT findings with instructions to monitor her pulse ox at home at rest and during exertion. Following completion of her R-CHOP, she was weaned off her prednisone with no rebound of symptoms.

A month after the prednisone taper, patient developed significant joint pain, trouble with ambulation, and was readmitted to the hospital. While admitted, she was started empirically on stress dose steroids for concern for cord compression and Central Nervous System (CNS) lymphoma recurrence. Magnetic Resonance Imaging (MRI) brain, cervical spine, thoracic spine, and lumbar spine showed no acute pathology, but symptoms did subjectively improve with steroids.

Further evaluation by Electromyography (EMG) showed multifactorial motor and sensory neuropathy with both axonal and demyelinating features. She was started on IVIG for 5 days with mild improvement of symptoms and was transferred to an acute rehabilitation center. Once at rehab, symptoms worsened again, and she was sent back to the hospital for plasmapheresis. However, given prolonged course of illness, decision was made by the patient and family to transition to comfort care.

## Discussion

We present a patient with drug induced lung injury thought to be secondary to rituximab and the complexity of managing a patient that needs this life saving medication while actively treating arising complications. Our patient was also on cyclophosphamide which can also cause lung injury that can happen in two distinct ways; either acute pneumonitis typically at 1–6 months or late onset fibrosis [7]. Given the hypersensitivity reaction during the infusion and acute onset after, rituximab was thought to be more likely the culprit rather than cyclophosphamide.

The frequency of RTX-ILD was initially thought to be 0.01–0.03%, but as it has become more commonly used this frequency has increased to 3.7–10% ^10^. In one case series looking at 121 causes of RTX-ILD, the mean number of cycles before pulmonary manifestations was 4.1 cycles with a median of 4 cycles. The length of cycles ranged from 2 to 4 weeks depending on disease process. They also found that the mean onset of disease after the last infusion was 30 days with a median of 15 days. Classification depends on onset and symptoms and can be hyperacute onset (within hours to 5 days) [8] or delayed-onset acute onset (within days)or rarely chronic(> 30 days) [1, 8]. While some studied describe Rituximab role in treating chronic immune mediated neuropathy including chronic inflammatory demyelinating polyradiculoneuropathy (CIDP), anti-myelin-associated glycoprotein (MAG) neuropathy, and autoimmune nodopath [1, 9]other smaller case series suggested possible worsening of Anti-MAG neuropathy [10]. However, neither looked at cancer patients in particular so it’s not applicable to our patient outside the known side effect of Rituximab being associated with peripheral neuropathy [11]. Interestingly, there are reports exploring progressive multifocal leukoencephalopathy(PML) due to reactivation of latent JC polyoma virus post rituximab [12].

The mechanism of RTX-ILD remains unknown, however several studies have demonstrated rapid lymphocyte lysis, complement activation, and Tumor Necrosis Factor (TNF) alpha release that occur after rituximab infusion [13]. The TNF alpha release has been postulated as a main component in the pathogenesis of ILD in porcine models. With TNF alpha release, there is an activation of cytokines, inflammatory mediators and angiogenic factors which could cause this ILD [14, 15]. Other studies postulated possible hypersensitivity reaction to the chimeric anti-CD20 antibody [8]. It’s worth considering the role of tumor microenvironment (TME). A study from 2018 explored the role of TME including immune cells, stromal cells, cytokines and other matrix component in tumor growth, progression and response to therapy [16].

Rituximab side effects are usually mild and include fevers, chills and rigors. Lung involvement is rare but classically presents with dry cough, hypoxemia, dyspnea and fever. CT findings can vary and include diffuse ground glass opacities sometimes with subpleural sparing, centrilobular nodules with occasionally consolidation, mosaic attenuation and pleural effusion [17, 18] Initial work up of RTX-ILD includes infection rule out, CT imaging, bronchoscopy and BAL, and in some cases lung biopsy. Given high risk for infections in patients undergoing active chemotherapy treatment, patients often undergo extensive infection work up to rule out viral and bacterial infections, atypical infections such as mycoplasma, and opportunistic infections [8]. PET-CT was performed in some cases and showed an early increase in tracer uptake [8]. Some cases showed alveolitis, pulmonary fibrosis, alveolar hemorrhage, pleural effusions [19, 20].

Bronchoscopy with bronchioalveolar lavage is occasionally performed, however there is no diagnostic yield specific to Rituximab ILD aside from ruling out infectious etiology. Cell counts after BAL showed predominant CD4 cells [8].In some cases, biopsy reveals pulmonary inflammation with features of lymphocyte predominance, desquamative alveolitis, histiocytosis, interstitial fibrosis, and non-necrotic granulomata that are overall non-specific findings suggestive of diffuse alveolar damage [1]. PFTs in patients with presumed RTX-ILD showed restrictive lung disease with decreased DLCO [1, 8, 17].

Management includes preferentially drug continuation but treatment with steroids could be considered after multidisciplinary discussion with experts [21]. In our patient, following the diagnosis of RTX-ILD, rituximab was immediately stopped, and she was started on high dose corticosteroids. In most cases, symptoms improve on corticosteroids, however in some cases symptoms can worsen when the corticosteroid dose was tapered. Given the curative intent of R-CHOP, rechallenge therapy has been attempted in select patients with success following completion of the high dose steroids without the need for a prolonged steroid maintenance therapy [8]. Alternatively, there are case series with successful rechallenge without the need for steroid, suggesting it is possible to continue therapy after initial drug induced injury [22]. These case series described highlight that is it is possible to continue R-CHOP without the need for prophylactic or maintenance steroids between the cycles.

Our case differs from those described above as our patient continued to receive rituximab along with prednisone for the rest of the cycles of R-CHOP. While the prednisone was successful in decreasing the hypoxia and lung manifestations of rituximab, some of the case series highlight that some patients might not have repeated lung disease progression even without the need for additional steroids. Following completion of her chemotherapy and as her prednisone was being tapered, our patient was diagnosed with a chronic inflammatory demyelinating polyneuropathy. High dose steroids have been used to treat demyelinating polyneuropathy, more specific chronic inflammatory demyelinating polyneuropathy. There are no reports in the literature of steroids causing idiopathic demyelinating neuropathy, however some reports have seen chronic inflammatory demyelinating polyradiculoneuropathy (CIDP) worsen after starting high dose steroids [11, 12].

Inconclusion, rituximab induced lung injury is a rare but series and potentially fatal. Early screening for symptoms, workup and prompt treatment with steroids is recommended.

## Data Availability

All data generated or analysed during this study are included in this published article [and its supplementary information files].

## Abbreviations

DLBCL: Diffuse Large B-cell Lymphoma
RTX-ILD: Rituximab associated Interstitial Lung disease
R-CHOP: Rituximab, Cyclophosphamide, Doxorubicin hydrochloride, Vincristine sulphate, and Prednisone (R-CHOP)
ARDS: Acute Respiratory Distress Syndrome
